# Ertapenem Once a Day Versus Piperacillin–Tazobactam Every 6 Hours for Treatment of Acute Pelvic Infections: A Prospective, Multicenter, Randomized, Double-Blind Study

**DOI:** 10.1155/S1064744903000048

**Published:** 2003

**Authors:** Subir Roy, Iliana Higareda, Edith Angel-Muller, Mahmoud Ismail, Caren Hague, Ben Adeyi, Gail L. Woods, Hedy Teppler

**Affiliations:** 1 Keck School of Medicine at USC Los Angeles CA USA; 2 Nuevo Hospital Civil Guadalajara Mexico; 3 Universidad Nacional de Colombia Bogota Colombia; 4 University of Chicago Chicago IL USA; 5 Merck Research Laboratories West Point PA USA; 6 Merck &Co., Inc. 10 Sentry Parkway BL 3-4 Blue Bell PA 19422 USA

## Abstract

*Objective:* To compare ertapenem therapy with piperacillin–tazobactam therapy for the management of acute pelvic infections.

*Methods:* In a multicenter, double-blind study, 412 women with acute pelvic infection were assigned to one of two strata, namely obstetric/postpartum infection or gynecologic/postoperative infection, and were then randomized to ertapenem, 1 g once a day, or piperacillin–tazobactam, 3.375 g every 6 hours, both administered intravenously.

*Results:* In total, 163 patients in the ertapenem group and 153 patients in the piperacillin–tazobactam group were clinically evaluable. The median duration of therapy was 4.0 days in both treatment groups. The most common single pathogen was *Escherichia coli* . At the primary efficacy endpoint 2–4 weeks post therapy, 93.9% of patients who received ertapenem and 91.5% of those who received piperacillin–tazobactam were cured (95% confidence interval for the difference, adjusting for strata, –4% to 8.8%), indicating that cure rates for both treatment groups were equivalent. Cure rates for both treatment groups were also similar when compared by stratum and severity of infection. The frequency and severity of drug-related adverse events were generally similar in both groups.

*Conclusions:*  In this study, ertapenem was as effective as piperacillin–tazobactam for the treatment of acute pelvic infection, was generally well tolerated, and had an overall safety profile similar to that of piperacillin–tazobactam.


*Infect Dis Obstet Gynecol 2003;11:27–37*

### Ertapenem once a day versus piperacillin–tazobactam every 6 hours for treatment of acute pelvic infections: a prospective, multicenter, randomized, double-blind study

**Subir Roy[1], Iliana Higareda[2], Edith Angel-Muller[3], Mahmoud Ismail[4], Caren Hague[5], Ben Adeyi[5], Gail L. Woods[5] and Hedy Teppler[5], for the Protocol 023 Study Group**

[1]*Keck School of Medicine at USC, Los Angeles, CA*
[2]*Nuevo Hospital Civil, Guadalajara, Mexico*
[3]*Universidad Nacional de Colombia, Bogota, Colombia*
[4]*University of Chicago, Chicago, IL*
[5]*Merck Research Laboratories, West Point, PA*

***Objective:*** To compare ertapenem therapy with piperacillin–tazobactam therapy for the management of acute pelvic infections.

***Methods:*** In a multicenter, double-blind study, 412 women with acute pelvic infection were assigned to one of two strata, namely obstetric/postpartum infection or gynecologic/postoperative infection, and were then randomized to ertapenem, 1 g once a day, or piperacillin–tazobactam, 3.375 g every 6 hours, both administered intravenously.

***Results:*** In total, 163 patients in the ertapenem group and 153 patients in the piperacillin–tazobactam group were clinically evaluable. The median duration of therapy was 4.0 days in both treatment groups. The most common single pathogen was *Escherichia coli*. At the primary efficacy endpoint 2–4 weeks post therapy, 93.9% of patients who received ertapenem and 91.5% of those who received piperacillin–tazobactam were cured (95% confidence interval for the difference, adjusting for strata, –4% to 8.8%), indicating that cure rates for both treatment groups were equivalent. Cure rates for both treatment groups were also similar when compared by stratum and severity of infection. The frequency and severity of drug-related adverse events were generally similar in both groups.

***Conclusions:*** In this study, ertapenem was as effective as piperacillin–tazobactam for the treatment of acute pelvic infection, was generally well tolerated, and had an overall safety profile similar to that of piperacillin–tazobactam.

Key words: ERTAPENEM; ACUTE PELVIC INFECTION; POSTPARTUM ENDOMYOMETRITIS; GYNECOLOGIC INFECTION

These data were presented in part at the 41st Interscience Conference on Antimicrobial Agents and Chemotherapy, Chicago, IL, December 2001.

Correspondence to: Gail L. Woods, MD, Merck & Co., Inc., 10 Sentry Parkway, BL 3-4, Blue Bell, PA 19422, USA. E-mail: gail_woods@merck.com

© 2003 The Parthenon Publishing Group

Acute soft tissue pelvic infections in women include several diagnoses that may be categorized as infections related to delivery and those which occur after gynecological surgery. Risk factors for acute pelvic infection are delivery by Cesarean section, hysterectomy or septic incomplete abortion. Although these procedures are often preceded or followed (for Cesarean section) by antimicrobial prophylaxis, the rate of infection may be as high as 20%[1]. Acute pelvic infections are usually polymicrobial. The major causal pathogens are those that comprise the normal vaginal flora, namely *Streptococcus agalactiae*, *Escherichia coli*, peptostreptococci, *Prevotella* spp., *Bacteroides* spp. and *Gardnerella vaginalis*. Antimicrobial regimens for the treatment of acute pelvic infection must therefore provide coverage against a broad spectrum of aerobic and anaerobic bacteria. Examples of effective regimens include combination therapy with an aminoglycoside and an agent such as metronidazole or clindamycin that provides anaerobic coverage, or monotherapy with agents that are dosed multiple times a day, such as cefoxitin, an extended-spectrum penicillin, or a β-lactam/β-lactamase inhibitor like piperacillin–tazobactam.

Ertapenem (Merck & Co., Inc., formerly MK-0826, Whitehouse Station, NJ) is a once-a-day parenteral β-lactam agent that can be used as monotherapy for the treatment of several community-acquired and mixed aerobic and anaerobic infections, including acute pelvic infections, complicated intra-abdominal, skin and urinary tract infections, and community-acquired pneumonia. This focused-spectrum carbapenem is highly active *in vitro* against many Gram-positive and Gram-negative aerobes and anaerobes that in general are associated with community-acquired infections, but has minimal activity against *Pseudomonas aeruginosa*, *Acinetobacter* spp. and enterococci[2,3]. The bacteria that are usually susceptible to ertapenem include *S. agalactiae* and many Enterobacteriaceae, other aerobic streptococci, and Gram-positive and Gram-negative anaerobes, which are the pathogens most commonly responsible for acute pelvic infections.

The objective of this study was to compare the efficacy, tolerability and safety of ertapenem 1 g once a day with those of piperacillin–tazobactam 3.375 g every 6 hours for the treatment of women with moderate to severe acute pelvic infection.

### SUBJECTS AND METHODS

### Patients

Females aged ≥ 16 years diagnosed with acute pelvic infection were eligible for inclusion in the study if they required at least 3 days of parenteral antimicrobial therapy and if the infection was caused by a pathogen susceptible to the study drugs. Criteria for acute pelvic infection included an oral temperature of > 38°C (or equivalent), white blood cell (WBC) count > 10 500/μl or > 10% immature granulocytes, and at least one of the following: pelvic, abdominal or uterine pain, cramping or tenderness, or an imaging study suggesting pelvic abscess or infection. Vaginal delivery, Cesarean section or gynecological surgery must have been performed between 24 hours and 1 month before enrollment. Patients with septic abortion could represent no more than 15% of the total enrollment.

Patients with any of the following were excluded from the study: pregnancy or lactation, history of serious allergy, hypersensitivity, or intolerance of study therapy (patients with a history of mild rash in response to β-lactams could be enrolled), pelvic inflammatory disease, tubo-ovarian abscess, postoperative abdominal wall infection, gynecological malignancy, any rapidly progressive disease, immunocompromising illness or therapy, AIDS (patients with HIV infection could be enrolled if they met the inclusion criteria), the need for concomitant antimicrobials (other than vancomycin, which was permitted for treatment of resistant Gram-positive pathogens in a mixed infection, or antifungal agents), acute hepatic failure, the need for peritoneal dialysis or hemodialysis, hypotension, a baseline pathogen resistant to either study drug, treatment with a systemic antimicrobial agent for ≥ 24 hours within 72 hours prior to admission to the study (unless failure of the prior regimen was documented), aspartate or alanine aminotransferase > 6 times the upper limit of normal (ULN), bilirubin or alkaline phosphatase > 3 times ULN, absolute neutrophil count ≤ 1000/μl, platelet concentration

< 75 000/μl, hematocrit < 20%, hemoglobin < 6 g/dl, or coagulation tests > 1.5 ULN.

### Study design and antimicrobial therapy

This prospective, double-blind (with sponsor blinding), randomized study was conducted from November 1998 to May 2000, at 47 sites, of which 30 sites (63.8%) were in the USA. In total, 17 sites enrolled 76.0% of the patients (≥ 10 patients/site). Written informed consent was obtained from all patients, and the institutional review board at each participating site approved the protocol and consent form. Eligible patients were stratified as follows. Stratum I consisted of patients with obstetric or postpartum infection (including septic abortion), and patients with gynecological or postoperative infection were included in Stratum II. Randomization in a 1 : 1 ratio (ertapenem: piperacillin–tazobactam) was performed using an allocation schedule that employed computer-generated random numbers.

Ertapenem 1 g once a day and piperacillin–tazobactam 3.375 g every 6 hours were given as intravenous (IV) infusions over a period of 30 minutes. The duration of treatment was determined by the investigator, and was usually 3–10 days. For patients with a creatinine clearance of < 30 ml/min/1.73 $m^2$, the dose of ertapenem was 500 mg once a day. The dose of piperacillin–tazobactam was adjusted to 2.25 g every 6 hours if the creatinine clearance was in the range 20–40 ml/min/1.73 $m^2$, and to 2.25 g every 8 hours if the creatinine clearance was < 20 ml/min/1.73 $m^2$. To ensure blinding, patients in the ertapenem group also received subsequent matching placebo infusions of 50 ml of normal saline every 6 hours. After at least 2 days of hospital infusion therapy, study therapy could be completed in the hospital, at a clinic or at home.

### Clinical assessments

Patients were evaluated at enrollment and daily thereafter while on parenteral study therapy. The clinical response was measured at the completion of parenteral therapy and 2–4 weeks post therapy – the test of cure (TOC) visit. The severity of the patient's infection was assessed prior to unblinding on the basis of prespecified criteria (patients who were hemodynamically unstable were not eligible for enrollment in the study). The infection was considered to be severe if the patient was bacteremic at baseline or had fever > 39°C. All other infections were considered to be of moderate severity. The clinical responses at the TOC visit were categorized as cure, presumptive cure (resolution of signs and symptoms of pelvic infection confirmed by telephone contact), failure (defined as death from acute pelvic infection, incomplete resolution or worsening of symptoms that required additional antimicrobial therapy, surgical intervention for pelvic infection > 24 hours after entry to the study, or surgical site infection that required additional antimicrobial therapy) or indeterminate (data not available for evaluation of efficacy). To be considered an evaluable failure, patients had to have received at least 48 hours of study antimicrobial therapy.

### Microbiological assessments

At enrollment, a specimen for aerobic and anaerobic culture was collected at surgery from the site of pelvic infection or, for patients with endometritis, from the endometrium by using a protected sampling device; high vaginal swabs were not acceptable. Subsequent pelvic cultures were only obtained if signs of ongoing or new pelvic infection were present. Blood cultures were performed if the patient had chills and/or a temperature of ≥ 39°C. All isolates were identified at the site laboratory, and aerobic pathogens were tested for *in-vitro* susceptibility to ertapenem and piperacillin–tazobactam following the guidelines of the National Committee for Clinical Laboratory Standards[4]. In addition, study sites outside the USA sent a duplicate clinical sample in an anaerobic transport tube (Anaerobe Systems, Morgan Hill, CA) to a central laboratory (R. M. Alden Research Laboratory, Santa Monica, CA) for culture and susceptibility testing of anaerobes.

The microbiological outcomes were categorized as eradication, presumptive eradication (no material available for culture in patients who were clinically cured; repeat cultures were required only in the context of clinical failure), persistence, persistence acquiring resistance, presumed persistence (culture not performed in patients considered to be clinical failures) or indeterminate (microbiological response could not be determined for any reason). Gram-positive pathogens treated with vancomycin were considered to have indeterminate microbiological outcomes. Treatment with vancomycin did not affect clinical assessability. Favorable microbiological outcomes were eradication and presumptive eradication.

### Populations for analysis

The treated population included all randomized patients who received at least one dose of study therapy. The clinical modified intent-to-treat (MITT) population consisted of treated patients who met the minimum disease definition. The clinically evaluable (per protocol) population was a subset of the clinical MITT population for whom information was sufficient to determine outcome at the TOC visit, and if baseline pathogens were present, at least one of these was susceptible to both study antimicrobials. Microbiologically evaluable patients were those clinically evaluable patients who had a baseline pathogen identified and a microbiological response assessed.

### Efficacy variables

The primary efficacy variable in this study was the clinical response assessment in the clinically evaluable population at the TOC visit. Additional efficacy assessments were the clinical response rates in the supportive clinical MITT population at the TOC visit and in the clinically evaluable population at completion of IV therapy, and the proportion of microbiologically evaluable patients with a favorable microbiological response at the TOC visit.

### Safety and tolerability assessment

All patients who received at least one dose of the study therapy were evaluated for safety and tolerability. Patients were monitored daily for adverse experiences during parenteral therapy and for 14 days thereafter. The intensity (mild, moderate or severe) of the adverse event and the likelihood of its being related to the study drug (definitely not, probably not, possibly, probably or definitely) were assessed by the investigator. The tolerability of each study drug at the local infusion site was evaluated daily by the investigator.

### Statistical analyses

The study was designed to test for equivalence in efficacy of the ertapenem and piperacillin–tazobactam clinically evaluable treatment groups. The sample size (a minimum of 150 evaluable patients per group) was calculated using Blackwelder's formula[5] and for the following values: alpha, 0.025; beta, 0.20; expected response rate in the comparator arm, 90%. Equivalence for this study was determined by calculation of the 95% (two-sided) confidence interval (CI) for the difference in response rates between the two treatment groups (ertapenem minus piperacillin–tazobactam). If the observed response rate in the comparator group was > 90%, for equivalence to be demonstrated, the CI of the difference had to contain zero and its lower limit could not be less than −10%. CIs about the difference were calculated using the normal approximation to the binomial distribution, and were adjusted for strata using the Cochran–Mantel–Haenzel approach[6]. The treatment × stratum interaction was investigated using the Breslow–Day test of homogeneity of odds ratios and the Gail–Simon test, if needed. An exploratory analysis using Kaplan–Meier curves was also performed to examine time to defervescence during therapy in clinically evaluable patients who were cured. No formal tests were performed based on baseline demographics or disease characteristics (e.g. severity).

### RESULTS

### Patients

The distribution of the study patients is summarized in Figure 1. In total, 38 patients signed a consent form but were not randomized. The most common reasons why patients were not randomized were failure to meet the criteria for diagnosis of acute pelvic infection (18 patients), withdrawal of consent (5 patients), and presence of a concurrent infection that would have interfered with

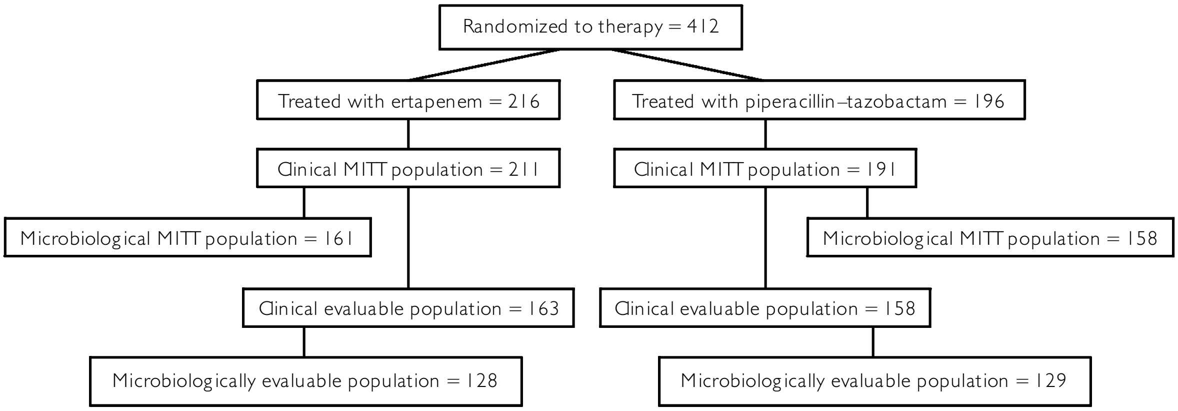

**Figure 1** Profile of patient enrollment. MITT, modified intent to treat evaluation of the response to study antimicrobial therapy (four patients). In total, 412 patients were randomized, 216 patients in the ertapenem group and 196 patients in the piperacillin– tazobactam group, of whom 163 subjects (75.5%) and 153 subjects (78.1%), respectively, were clinically evaluable. The most common reasons why patients were not clinically evaluable were assessments outside the protocol-defined follow-up period, and inadequate or inappropriate courses of study therapy.

The baseline demographics of the patients who signed a consent form but were not randomized appeared to be comparable to those of the randomized population (data not shown). The baseline demographics and disease characteristics of the two treatment groups in the randomized and clinically evaluable populations were generally similar (Table 1). The majority of patients in both populations were in stratum I (obstetric/ postpartum infection), and the most common diagnosis at entry was endomyometritis (present in approximately 75% of patients in both populations). In approximately 25% of patients the infection was rated as severe.

### Therapy

The dosage of study drug was adjusted for renal insufficiency in four patients (one in the ertapenem group and three in the piperacillin–tazobactam group), all of whom were considered not to be clinically evaluable for reasons other than elevated creatinine clearance values. The duration of study therapy in the treated and clinically evaluable patients was comparable in the ertapenem and piperacillin–tazobactam treatment groups (Table 1). The median duration was 4 days in each treatment group of both populations. Six patients received vancomycin for resistant Gram-positive pathogens; none of them met the criteria for inclusion in the clinically evaluable population.

### Baseline microbiology

Of the clinically evaluable patients, 128 patients (78.5%) in the ertapenem group and 129 patients (84.3%) in the piperacillin–tazobactam group had at least one pathogen isolated at baseline. In total, 93 microbiologically evaluable patients (72.7%) who received ertapenem and 93 patients (72.1%) who received piperacillin–tazobactam had polymicrobic infections. The distribution of the pathogens in each treatment group of the microbiologically evaluable population and their susceptibility profiles were comparable. Anaerobes accounted for 60.8% (257/423) of the isolates in the ertapenem group and 58.6% (238/406) of the isolates in the piperacillin–tazobactam group. The most common single isolate in each group was *E. coli* (41 (9.7%) and 39 (9.6%) in the ertapenem and piperacillin–tazobactam groups, respectively). Most isolates were susceptible to both study drugs, with the exception of enterococci, which were often intermediate or resistant to ertapenem. In total, ten microbiologically evaluable patients in the ertapenem group (five with endometritis and five with septic abortion) and six patients in the

**Table 1** Baseline characteristics and therapy of randomized and clinically evaluable patients with acute pelvic infection, by treatment group

| | *Randomized*[a] | | *Clinically evaluable* | |
|---|---|---|---|---|
| *Characteristic* | *Ertapenem* (*n* = 216) | *Pip–Taz* (*n* = 196) | *Ertapenem* (*n* = 163) | *Pip–Taz* (*n* = 153) |
| *Ethnicity (percent)* | | | | |
| Caucasian | 46 (21.3) | 41 (20.9) | 34 (20.9) | 32 (20.9) |
| Black | 66 (30.6) | 52 (26.5) | 42 (25.8) | 38 (24.8) |
| Hispanic | 73 (33.8) | 69 (35.2) | 57 (35.0) | 55 (35.9) |
| Mestizo | 29 (13.4) | 32 (16.3) | 28 (17.2) | 26 (17.0) |
| Other ethnic group | 2 (0.9) | 2 (1.0) | 2 (1.2) | 2 (1.4) |
| *Mean age* (± SD) (years) | 25.4 (± 7.5) | 27.0 (± 8.9) | 25.7 (± 7.6) | 27.6 (± 9.2) |
| *Stratum*[b] *(percent)* | | | | |
| Obstetric/postpartum infection | 181 (83.8) | 169 (86.2) | 136 (83.4) | 132 (86.3) |
| Vaginal delivery | 79 (36.6) | 66 (33.7) | 60 (36.8) | 50 (32.7) |
| Cesarean section | 85 (39.4) | 87 (44.4) | 60 (36.8) | 68 (44.4) |
| Gynecological/postoperative infection | 34 (15.7) | 27 (13.8) | 27 (16.6) | 21 (13.7) |
| *Severe infection (percent)* | 60 (27.8) | 48 (24.5) | 42 (25.8) | 35 (22.9) |
| *Antimicrobial prophylaxis given*[c] *(percent)* | 89 (41.2) | 93 (47.4) | 65 (39.9) | 68 (44.4) |
| *Diagnosis at entry (percent)* | | | | |
| Endomyometritis | 164 (75.9) | 148 (75.5) | 120 (73.6) | 115 (75.2) |
| Septic abortion | 22 (10.2) | 23 (11.7) | 20 (12.3) | 19 (12.4) |
| Pelvic cellulitis | 7 (3.2) | 10 (5.1) | 6 (3.7) | 9 (5.9) |
| Pelvic abscess | 8 (3.7) | 7 (3.6) | 4 (2.5) | 5 (3.3) |
| Parametritis | 7 (3.2) | 6 (3.1) | 6 (3.7) | 4 (2.6) |
| Other | 7 (3.2) | 2 (1.0) | 7 (4.3) | 1 (0.7) |
| *Median days on therapy* (range) | 4.0 (1–13) | 4.0 (1–12) | 4.0 (2–12) | 4.0 (3–12) |

[a]Treated patients for days on therapy. For ertapenem, *n* = 214; for piperacillin–tazobactam, *n* = 192; [b]One patient in the ertapenem group was not included in the stratification counts because no primary diagnosis information was provided and the patient received no therapy; [c]Includes prophylaxis for surgical procedures and obstetric conditions; Pip–Taz, piperacillin–tazobactam; SD, standard deviation piperacillin–tazobactam group (two with endometritis, two with septic abortion and two with pelvic cellulitis) were bacteremic at baseline. In patients who received ertapenem, causal pathogens were *E. coli* (*n* = 6) and one each of the following: *Listeria monocytogenes*, *Enterobacter cloacae*, *Klebsiella pneumoniae* and *Prevotella loescheii*. In those who received piperacillin–tazobactam, causal pathogens were *E. coli* (*n* = 2) and one each of the following: *E. cloacae*, *Streptococcus pyogenes*, *Peptostreptococcus asaccharolyticus*, *Streptococcus* spp., *Arcanobacterium bernardiae* and *Corynebacterium* spp. (the latter two organisms were recovered from one patient).

### Efficacy

At the primary efficacy endpoint, 93.9% of the clinically evaluable patients in the ertapenem group and 91.5% of those in the piperacillin–tazobactam group were cured (95% CI for the difference, adjusting for strata, −4.0% to 8.8%), indicating equivalence between the two treatments. Clinical success rates at the TOC assessment

**Table 2** Cure rates in clinically evaluable patients, by stratum or subgroup

| | *Ertapenem* | | *Piperacillin–tazobactam* | |
|---|---|---|---|---|
| *Stratum/subgroup* | *n/m* | *% response (95% CI)* | *n/m* | *% response (95% CI)* |
| *At DCIV* | | | | |
| Stratum | | | | |
| Obstetric/postpartum infection | 130/137 | 94.9 (91.2, 98.6) | 122/132 | 92.4 (87.9, 97.0) |
| Gynecological/postoperative infection | 25/26 | 96.2 (88.6, 100) | 19/21 | 90.5 (77.6, 100) |
| Overall | 155/163 | 95.1 (91.8, 98.4) | 141/153 | 92.2 (87.9, 96.4) |
| *At test of cure* | | | | |
| Stratum | | | | |
| Obstetric/postpartum infection | 129/137 | 94.2 (90.2, 98.1) | 121/132 | 91.7 (86.9, 96.4) |
| Gynecological/postoperative infection | 24/26 | 92.3 (81.9, 100) | 19/21 | 90.5 (77.6, 100) |
| Severity | | | | |
| Moderate infection | 113/121 | 93.4 (87.4, 97.1) | 110/118 | 93.2 (87.1, 97.0) |
| Severe infection | 40/42 | 95.2 (83.8, 99.4) | 30/35 | 85.7 (69.7, 95.2) |
| Diagnosis | | | | |
| Endomyometritis | 111/120 | 92.5 (86.2, 96.5) | 104/115 | 90.4 (83.5, 95.1) |
| Septic abortion | 20/20 | 100 (83.2, 100) | 19/19 | 100 (82.4, 100) |
| Overall | 153/163 | 93.9 (90.2, 97.6) | 140/153 | 91.5 (87.1, 95.9) |

n/m, ratio of number of patients cured/number of patients with assessment; CI, confidence interval; DCIV, discontinuation of IV therapy in the supportive clinical MITT analysis, which included 97.6% of the randomized patients (211 patients treated with ertapenem and 191 patients treated with piperacillin–tazobactam), were 85.9% in the ertapenem group and 88.0% in the piperacillin–tazobactam group. This reflects the more conservative approach in the MITT outcome assessment, in which patients with inadequate information or indeterminate outcomes were considered to be cases of treatment failure. The difference (95% CI) between the response rates in the two clinical MITT groups of −2.1% (−9.2% to 5.0%) indicates that the two treatment groups were similar, which is consistent with the results of the primary efficacy analysis. Among patients with postpartum endomyometritis, cure rates were higher in those who had vaginal delivery (ertapenem, 95.0% or 57/60; piperacillin–tazobactam, 96.0% or 48/50) than in those who underwent Cesarean section (ertapenem, 91.7% or 55/60; piperacillin–tazobactam, 86.8% or 59/68).

Table 2 shows the cure rates for clinically evaluable patients in the two treatment groups by stratum (at completion of study therapy and at the TOC assessment), severity of infection and primary diagnosis. More than 90% of the patients in each stratum or subgroup of both treatment groups had a favorable clinical response, with one exception. In patients with severe infection, cure rates were 95.2% for those in the ertapenem group and 85.7% for those in the piperacillin–tazobactam group. Statistical analyses within subgroups, such as patients with severe infection, were not specified a priori and therefore were not performed. The most common reason why patients were considered to have clinical failure at the TOC assessment was persistent, unresolved or worsening infection (7/10 or 70% of patients treated with ertapenem and 7/13 or 53.8% of patients treated with piperacillin–tazobactam). Other reasons were surgical intervention > 24 hours after study entry (1/10 or 10% of patients in the ertapenem group and 4/13 or 30.8% of those in the piperacillin–tazobactam group), and surgical site infection requiring additional antimicrobial therapy (2/10 or 20% of patients in the ertapenem group and 2/13 or 15.4% of patients in the piperacillin–tazobactam group).

**Table 3** Eradication/presumed eradication rates[a] at test of cure, by baseline pathogen isolated from the site of the pelvic infection and/or from blood

| Organism | Proportion (%) eradicated by: Ertapenem | Piperacillin–tazobactam |
|---|---|---|
| *Listeria monocytogenes* | 1/1 (100) | NI |
| Other aerobic GPB | 4/5 (80.0) | 2/2 (100) |
| *Staphylococcus aureus* | 9/9 (100) | 16/16 (100) |
| Other staphylococci | 17/17 (100) | 15/16 (93.8) |
| *Streptococcus agalactiae* | 11/11 (100) | 16/16 (100) |
| Other streptococci | 25/27 (92.6) | 18/21 (85.7) |
| Enterococci | 23/23 (100) | 30/31 (96.8) |
| Other aerobic GPC | 3/3 (100) | NI |
| *Escherichia coli* | 37/41 (90.2) | 36/39 (92.3) |
| Other Enterobacteriaceae | 20/22 (90.9) | 20/20 (100) |
| Nonfermentative GNB | 3/4 (75.0) | 4/4 (100) |
| Other aerobic GNB | NI | 2/2 (100) |
| *Neisseria* spp. | 1/1 (100) | 1/1 (100) |
| *Clostridium* spp. | 11/11 (100) | 10/10 (100) |
| Other anaerobic GPB | 7/7 (100) | 11/13 (84.6) |
| *Peptostreptococcus* spp. | 81/83 (97.6) | 76/82 (92.7) |
| Other anaerobic GPC | NI | 1/1 (100) |
| *Bacteroides fragilis* group | 30/30 (100) | 29/32 (90.6) |
| *Bacteroides fragilis* | 15/15 (100) | 19/20 (95.0) |
| Other members of *B. fragilis* group | 15/15 (100)[b] | 10/12 (83.3)[c] |
| *Fusobacterium* spp. | 15/15 (100) | 9/11 (81.8) |
| *Porphyromonas* spp. | 26/26 (100) | 22/23 (95.7) |
| *Prevotella* spp. | 54/54 (100) | 46/50 (92.0) |
| Other anaerobic GNB | 30/30 (100) | 14/14 (100) |
| Anaerobic GNC | 3/3 (100) | 2/2 (100) |

NI, no isolates; GPB, Gram-positive bacilli; GPC, Gram-positive cocci; GNB, Gram-negative bacilli; GNC, Gram-negative cocci.
[a]Repeat endometrial cultures were not collected from patients who were cured; [b]*B. thetaiotaomicron* (4/4), *B. vulgatus* (4/4), *B. distasonis* (3/3), *B. uniformis* (2/2); [c]*B. thetaiotaomicron* (5/6), *B. vulgatus* (1/2), *B. distasonis* (4/4)

The time to resolution of baseline clinical signs and symptoms was similar in each treatment group. Patient signs and symptoms, assessed daily, had resolved in 134 of 158 patients (84.8%) in the ertapenem group and in 124 of 147 patients (84.4%) in the piperacillin–tazobactam group at completion of study therapy, and in 141 of 154 patients (91.6%) and 132 of 146 patients (90.4%), respectively, at the TOC assessment. In addition, the Kaplan–Meier curves for time to defervescence in the clinically evaluable patients who were cured appeared to be similar for ertapenem and piperacillin–tazobactam (data not shown). Defervescence was prompt in each treatment group, as 90% of patients were afebrile, regardless of study therapy, by study day 2.

The proportion of microbiologically evaluable patients who at the TOC assessment had a favorable overall microbiological response (i.e. all baseline pathogens were eradicated or presumed to be eradicated) was 93.7% in the ertapenem group and 93.8% in the piperacillin–tazobactam group. Bacterial eradication/presumed eradication rates at the TOC assessment, shown by the baseline pathogen in Table 3, were generally similar in both treatment groups. Of the patients with an unfavorable microbiological response, persistence was documented for only one pathogen (*E. coli*). For all other pathogens, persistence was presumed based on the clinical outcome.

### Safety and local tolerability

In total, 214 patients in the ertapenem group and 192 patients in the piperacillin–tazobactam group received at least one dose of study parenteral therapy and were evaluated for adverse experiences. During parenteral therapy and for 14 days thereafter, one or more drug-related adverse experiences were reported for 48 patients (22.4%) in the ertapenem group and 43 patients (22.4%) in the piperacillin–tazobactam group. The most common drug-related adverse events were mild gastrointestinal symptoms. Eight patients (3.7%) who were treated with ertapenem and four patients (2.1%) who were treated with piperacillin–tazobactam had diarrhea. Vomiting occurred in five patients (2.3%) in the ertapenem group and four patients (2.1%) in the piperacillin–tazobactam group, and six patients (2.8%) who received ertapenem and three patients (1.6%) who received piperacillin–tazobactam experienced nausea. Five patients (2.3%) who received ertapenem and five patients (2.6%) who received piperacillin–tazobactam had headache.

One patient in the ertapenem group discontinued study therapy due to elevation of the serum creatinine levels and renal insufficiency on study day 3. The investigator considered this to be a serious adverse event that was probably related to study therapy; concomitant medications included ibuprofen. Antimicrobial therapy was changed from ertapenem to ampicillin plus clindamycin, both of which were discontinued on study day 5 because the creatinine concentration continued to rise. On study day 6, a nephrologist was consulted. It was this consultant's opinion that the patient had acute interstitial nephritis or an acute renal insult, most probably caused by ibuprofen use. The serum creatinine concentration had returned to normal by study day 10. Study therapy was discontinued in three additional patients in the ertapenem group, due to an overdose of study drug resulting from a pharmacy dispensing error. No adverse effects associated with the overdose were reported. None of the patients in the piperacillin–tazobactam group had study therapy discontinued because of a drug-related adverse experience.

Drug-related laboratory adverse experiences during parenteral therapy and for 14 days thereafter were reported in 26 patients (13.2%) in the ertapenem group and 29 patients (15.7%) in the piperacillin–tazobactam group. The most common drug-related adverse laboratory events were thrombocytosis (19/190 or 10.0% of patients in the ertapenem group and 20/178 or 11.2% of patients in the piperacillin–tazobactam group) and elevation of liver enzymes as follows: alkaline phosphatase, eight patients (4.6%) in the ertapenem group and four patients (2.4%) in the piperacillin–tazobactam group; alanine aminotransferase, six patients (3.3%) and three patients (1.8%), respectively; aspartate aminotransferase, six patients (3.2%) and two patients (1.1%), respectively.

In total, 26 patients (12.2%) in the ertapenem group and 24 patients (12.5%) in the piperacillin–tazobactam group experienced reactions of moderate to severe intensity at the local infusion site during parenteral therapy. The most common symptom in both treatment groups was pain, followed by tenderness and induration.

### DISCUSSION

In this multicenter clinical trial, ertapenem therapy, 1 g once a day, was highly effective for treatment of women with acute pelvic infection, including those with severe infection, and was equivalent to treatment with piperacillin–tazobactam given every 6 hours. In addition, the time to defervescence and resolution of other signs and symptoms was similar for both agents. Approximately 94% of clinically evaluable patients treated with ertapenem were cured, compared with 92% of those who were treated with piperacillin–tazobactam. These cure rates are similar to or higher than those reported in previous studies of patients with acute pelvic infection[7–11], and they are within the range of the expected cure rate (i.e. approximately 90%) when evaluating a new anti-infective drug for treatment of acute pelvic infection[12]. As predicted, the success rates in patients with septic abortion were excellent (100%) in both treatment groups.

Ertapenem is highly active *in vitro* against many Gram-positive and Gram-negative aerobic, facultative and anaerobic bacteria that are generally associated with infections acquired in the community[2,3]. In this study, aerobic streptococci, Enterobacteriaceae, peptostreptococci and anaerobic Gram-negative anaerobes, over 99% of which were susceptible to ertapenem and piperacillin–tazobactam, accounted for approximately 80% of all isolates from microbiologically evaluable patients in both treatment groups. Other investigators have also found that these same isolates are responsible for most acute pelvic infections[7–11]. *In vitro*, ertapenem has limited activity against *P. aeruginosa* and enterococci. *P. aeruginosa* is rarely recovered from patients with acute pelvic infection. In this study, there were no isolates in either treatment group. However, *Enterococcus* is occasionally encountered in cultures from such patients. In this study, 23 patients who were treated with ertapenem had a polymicrobial infection that included *Enterococcus*, and all of them had a favorable clinical and microbiological outcome at the TOC assessment. This suggests that in polymicrobial acute pelvic infections, additional specific anti-enterococcal therapy is not required. Similarly, many physicians consider enterococci to be part of the normal vaginal flora rather than pathogens, unless they are recovered in the presence of a prosthesis[13].

For acute pelvic infections, once-a-day dosing with an antimicrobial agent such as ertapenem offers several potential advantages over other common treatment regimens that require multiple daily doses and/or a combination of antimicrobial agents. Such advantages may include facilitation of outpatient therapy (either intravenously or by intramuscular injection), decreased treatment costs[14] and a potential reduction in the medication error rate in hospitalized patients[15].

The overall safety profile and tolerability of ertapenem in this study were similar to those of piperacillin–tazobactam. Mild gastrointestinal symptoms were the most frequently reported drug-related clinical adverse events for both agents. The most common drug-related laboratory adverse events for both drugs were thrombocytosis and mild to moderate elevation of liver enzymes, both of which were transient and without clinical consequence.

In summary, ertapenem 1 g once a day was highly effective both clinically and microbiologically in the treatment of women with moderate to severe acute pelvic infection. Ertapenem therapy was as effective as therapy with piperacillin–tazobactam, and had a comparable overall safety and tolerability profile.

### ACKNOWLEDGEMENTS

This study was supported by Merck & Co., Inc.

Members of the Protocol 023 Study Group include the following: Joseph Apuzzio, Newark, NJ; David Baker, Stony Brook, NY; Guy Benrubi, Jacksonville, FL; Ashwin Chatwani, Philadelphia, PA; Dean Coonrod, Phoenix, AZ; Lawrence Devoe, Augusta, GA; Patrick Duff, Gainsville, FL; Stanley Gall, Louisville, KY; Larry Gilstrap, Houston, TX; Alice Goepfert, Birmingham, AL; Phillip Greig, Greenville, SC; Mark Harrison, Berrien Center, MI; David Hemsell, Dallas, TX; Peter Heyl, Norfolk, VA; Mahmoud Ismail, Chicago, IL; Abner Korn, San Francisco, CA; William Ledger, New York, NY; Maurizio Maccato, Houston, TX; Everett Magann, Jackson, MS; Mark Martens, Minneapolis, MN; James McGregor, Denver, CO; S. Gene McNeeley, Detroit, MI; Lynnae Millar, Honolulu, HA; Susan Mou, Rochester, NY; Edward Newton, Greenville, NC; William O'Brien, Tampa, FL; R. Lamar Parker, Winston-Salem, NC; Subir Roy, Los Angeles, CA; Thomas Stovall, Memphis, TN; Richard Sweet, Pittsburgh, PA; Kristi Van Nostrand, Oklahoma City, OK; Amadeo Aldini, Buenos Aires, Argentina; Pedro Lipszye, Buenos Aires, Argentina; Osvaldo Malafaia, Curitiba Parana', Brazil; Kay Sander, San Jose, Costa Rica; Edith Angel-Muller, Bogota, Colombia; Vincente Carmona, Bogota, Colombia; Antonio Ciudad, Lima, Peru; Juan Trelles, Lima, Peru; Denis Jacob, Paris, France; Prof. Philippe Judlin, Nancy, France; Eric de Jonge, Pretoria, South Africa; Thembeni Misibi, Johannesburg, South Africa; Bareno Lindeque, Pretoria, South Africa; Alexander Davidov, Moscow, Russia; Galina Saveliea, Moscow, Russia; Enrique Garcia-Lara, Mexico City, Mexico; Iliana Higareda, Guadalajara, Jalisco, Mexico.

### REFERENCES

1. Faro S. Antibiotic usage in pelvic infections: an overview. *J Reprod Med* 1988;33(Suppl. 6):566–70
2. Livermore DM, Carter MW, Bagel S, *et al. In-vitro* activities of ertapenem (MK-0826) against recent clinical bacteria collected in Europe and Australia. *Antimicrob Agents Chemother* 2001;45:1860–67
3. Fuchs PC, Barry AL, Brown SD. *In-vitro* activities of ertapenem (MK-0826) against clinical bacterial isolates from 11 North American medical centers. *Antimicrob Agents Chemother* 2001;45:1915–18
4. National Committee for Clinical Laboratory Standards. *Performance Standard for Antimicrobial Disk Susceptibility Tests: Approved Standard*. NCCLS Document M2-A6. Wayne, PA: National Committee for Clinical Laboratory Standards, 1997
5. Blackwelder WC. 'Proving the null hypothesis' in clinical trials. *Control Clin Trials* 1982;3:345–53
6. Cochran WG. Some methods for strengthening the common Chi-square tests. *Biometrics* 1954;10: 417–51
7. Crombleholme WR, Ohm-Smith M, Robbie MO, *et al.* Ampicillin/sulbactam versus metronidazole–gentamicin in the treatment of soft tissue pelvic infections. *Am J Obstet Gynecol* 1987;156:507–12

8. Hemsell DL, Wendel GD, Gall SA, *et al.* Multicenter comparison of cefotetan and cefoxitin in the treatment of acute obstetric and gynecologic infections. *Am J Obstet Gynecol* 1988;158:722–7
9. Apuzzio JJ, Stankiewicz R, Ganesh V, *et al.* Comparison of parenteral ciprofloxacin with clindamycin–gentamicin in the treatment of pelvic infection. *Am J Med* 1989;87(Suppl. 5A): S148–51
10. Sweet RL, Roy S, Faro S, *et al.* Piperacillin and tazobactam versus clindamycin and gentamicin in the treatment of hospitalized women with pelvic infection. *Obstet Gynecol* 1994;83:280–6
11. Roy S, Koltun W, Chatwani A, *et al.* Treatment of acute gynecologic infections with trovafloxacin. *Am J Surg* 1998;176(Suppl. 6A):S67–73
12. Hemsell DL, Solomkin JS, Sweet R, *et al.* Evaluation of new anti-infective drugs for the treatment of acute pelvic infections in hospitalized women. *Clin Infect Dis* 1992;15 (Suppl. 1):S43–52
13. Gall S. Therapeutic dilemmas in the treatment of pelvic infections. *J Reprod Med* 5(Suppl.): 1091–4
14. Foran RM, Brett JL, Wulf PH. Evaluating the cost impact of intravenous antibiotic dosing frequencies. *DICP* 1991;25:546–52
15. Nettleman MD, Bock MJ. The epidemiology of missed medication doses in hospitalized patients. *Clin Perform Qual Health Care* 1996;4: 148–53

Received 06/26/02; Accepted 10/14/02

## References

[PDF_00975] Blackwelder W. C. (1982). "Proving the null hypothesis" in clinical trials.. Control Clin Trials.

[PDF_00980] Crombleholme W. R., Ohm-Smith M., Robbie M. O., DeKay V., Sweet R. L. (1987). Ampicillin/sulbactam versus metronidazole-gentamicin in the treatment of soft tissue pelvic infections.. Am J Obstet Gynecol.

[PDF_00959] Faro S. (1988). Antibiotic usage in pelvic infections. An overview.. J Reprod Med.

[PDF_01008] Foran R. M., Brett J. L., Wulf P. H. (1991). Evaluating the cost impact of intravenous antibiotic dosing frequencies.. DICP.

[PDF_00965] Fuchs P. C., Barry A. L., Brown S. D. (2001). In vitro activities of ertapenem (MK-0826) against clinical bacterial isolates from 11 North American medical centers.. Antimicrob Agents Chemother.

[PDF_01003] Hemsell D. L., Solomkin J. S., Sweet R., Tally F., Bartlett J. G. (1992). Evaluation of new anti-infective drugs for the treatment of acute pelvic infections in hospitalized women. Infectious Diseases Society of America and the Food and Drug Administration.. Clin Infect Dis.

[PDF_00987] Hemsell D. L., Wendel G. D., Gall S. A., Newton E. R., Gibbs R. S., Knuppel R. A., Lane T. W., Sweet R. L. (1988). Multicenter comparison of cefotetan and cefoxitin in the treatment of acute obstetric and gynecologic infections.. Am J Obstet Gynecol.

[PDF_00961] Livermore D. M., Carter M. W., Bagel S., Wiedemann B., Baquero F., Loza E., Endtz H. P., van Den Braak N., Fernandes C. J., Fernandes L. (2001). In vitro activities of ertapenem (MK-0826) against recent clinical bacteria collected in Europe and Australia.. Antimicrob Agents Chemother.

[PDF_01014] Nettleman M. D., Bock M. J. (1996). The epidemiology of missed medication doses in hospitalized patients.. Clin Perform Qual Health Care.

[PDF_00996] Sweet R. L., Roy S., Faro S., O'Brien W. F., Sanfilippo J. S., Seidlin M. (1994). Piperacillin and tazobactam versus clindamycin and gentamicin in the treatment of hospitalized women with pelvic infection. The Piperacillin/tazobactam Study Group.. Obstet Gynecol.

